# Cell Signaling Experiments Driven by Optical Manipulation

**DOI:** 10.3390/ijms14058963

**Published:** 2013-04-25

**Authors:** Francesco Difato, Giulietta Pinato, Dan Cojoc

**Affiliations:** 1 Department of Neuroscience and Brain Technologies (NBT), Italian Institute of Technology (IIT), Via Morego 30, Genoa 16163, Italy; E-Mail: francesco.difato@iit.it; 2 Centre for Biomedical Sciences and Engineering, University of Nova Gorica, Vipavska 13, Rožna Dolina, Nova Gorica SI-5000, Slovenia; E-Mail: giulietta.pinato@ung.si; 3 Optical Manipulation Lab, Institute of Materials–National Research Council (IOM-CNR), Area Science Park-Basovizza, S.S. 14, km 163.5, Trieste 34149, Italy

**Keywords:** cell signaling, optical manipulation, optical force probing, local delivery, laser nano-ablation, force spectroscopy, mechanotransduction

## Abstract

Cell signaling involves complex transduction mechanisms in which information released by nearby cells or extracellular cues are transmitted to the cell, regulating fundamental cellular activities. Understanding such mechanisms requires cell stimulation with precise control of low numbers of active molecules at high spatial and temporal resolution under physiological conditions. Optical manipulation techniques, such as optical tweezing, mechanical stress probing or nano-ablation, allow handling of probes and sub-cellular elements with nanometric and millisecond resolution. PicoNewton forces, such as those involved in cell motility or intracellular activity, can be measured with femtoNewton sensitivity while controlling the biochemical environment. Recent technical achievements in optical manipulation have new potentials, such as exploring the actions of individual molecules within living cells. Here, we review the progress in optical manipulation techniques for single-cell experiments, with a focus on force probing, cell mechanical stimulation and the local delivery of active molecules using optically manipulated micro-vectors and laser dissection.

## 1. Introduction

Cells continuously communicate with each other through chemical, electrical and mechanical signals. Cell signaling involves complex transduction mechanisms in which information released by nearby cells or extracellular cues are transmitted to the cell, regulating fundamental cellular activities [1]. Stimuli (e.g., hormones, neurotransmitters or growth factors) acting on cell-surface receptors relay information through intracellular signaling pathways that include a number of components. Signaling usually begins with the activation of transducers that, by amplification mechanisms, trigger internal messengers that either act locally or diffuse throughout the cell. These messengers then engage sensors that are coupled to effectors that activate cellular responses. Cell signaling is a dynamic process comprising “on” mechanisms by which information flows down the signaling pathway in response to external stimuli, and it is opposed by “off” mechanisms that are responsible for switching off the signaling system when the external stimuli are withdrawn. Given the complexity of cell signaling, studying its mechanisms requires experimental approaches able to address cell stimulation with high spatial and temporal resolution.

Most cellular research calls for optical microscopy, either to perform cell imaging or/and for visualizing sample manipulation. Concerning imaging, recently developed super-resolution methods have overcome the diffraction limit barrier and bio-samples can hence be imaged at a resolution of tens of nm [2]. Most of these techniques can be implemented using a normal inverted microscope, such as those used in most cell biology laboratories. Moreover, the advances in laser technology make integrating optical manipulation tools into the same platform possible.

Optical manipulation includes three-dimensional particle trapping, manipulation by means of optical tweezers (OT), and photolysis of liposomes and cell compartments by laser ablation. Since their discovery [3], OT have been continuously developed and have found many applications in the physical, biological and chemical sciences. In fact, with the very first proof of principle using silica microbeads, Ashkin demonstrated the possibility of optically trapping and manipulating viruses and bacteria [4], as well as single cells [5] and even sub-organelles, using infrared (IR) laser beams [6]. Manipulation with infrared laser beams was proven to not damage cells even if the levels of intensity were high (tens of MW/cm^2^, *i.e.*, power higher than 100 mW focused on a spot of about 1 μm^2^). Another important achievement was the measurement of the forces generated by organelle transport *in vivo* [7]. A comprehensive outlook on the development of OT technology and its applications in different fields can be found in several very good reviews [8–13]. The use of laser microsurgery in cell and developmental biology was introduced during the same period as OT as a result of investigations into the potential applications of lasers [14]. The laser microsurgery technique has evolved in parallel with that of OT [15], and it can be implemented jointly with optical trapping on the same microscope platform for single-cell signaling experiments.

**Figure 1:**
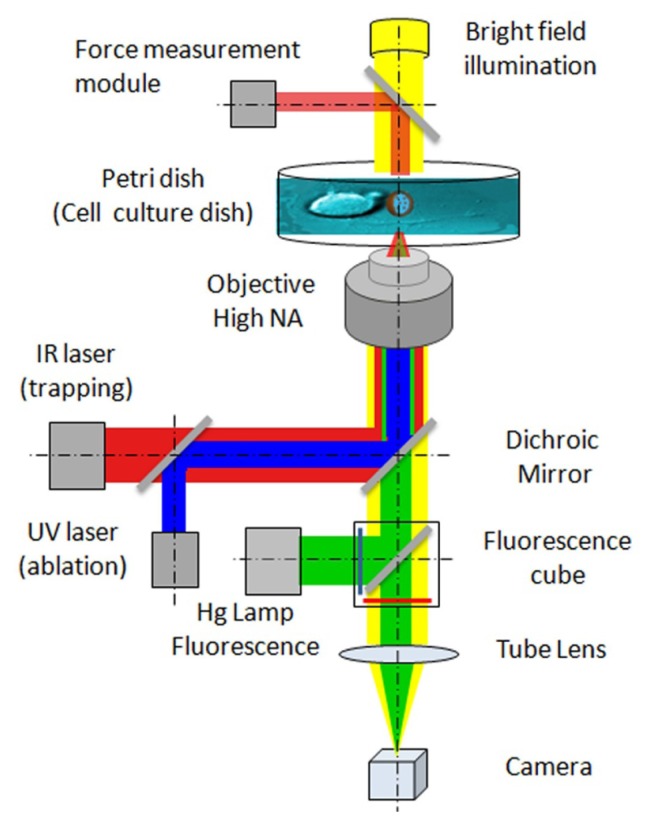
Optical manipulation for single-cell experiments: IR laser trapping, UV laser ablation and force measurement modules implemented on an inverted optical microscope (adapted from [16]).

The implementation of optical manipulation on a standard optical microscope and some examples of the typical conditions for living cell experiments are schematized in Figure 1. The cells are plated in a Petri dish and imaged through an optical microscope using bright field (yellow beam) illumination or fluorescence excitation/emission (green beam). Additional laser beams can be easily introduced into the optical path of a modern microscope with infinite conjugated optics (*i.e.*, the objective creates an intermediate image at infinity, and the tube lens focuses the final image on the camera) because different optical components (e.g., a dichroic mirror in Figure 1) can be hosted between the objective and the tube lens. An IR laser beam (red beam) is introduced for particle trapping and manipulation. Continuous wave laser beams with IR wavelengths between 900 and 1100 nm are typically used because they do not damage cells. An ultra-violet (UV) laser beam can also be inserted for laser ablation of cellular components or liposomal carriers. UV (350 nm) laser beams with ns–ps pulses or IR (800 nm) laser beams with fs–ps pulses are usually used for laser ablation.

Cell signaling investigations are normally performed in bulk by micropipetting active molecules into the medium to change the biochemical environment for all of the cells and then observing only one or a few of them. Such an experimental approach has several limitations. Bulk stimulation of the cells might saturate the receptor-activated responses, the time required for the molecules to reach their target cannot be defined and thus there is no synchronization between different activated cell signaling pathways, and large amounts of active molecules are required. Moreover, neighboring cells could respond to the bulk stimulation by exocytosis of active molecules, interfering with the specific process under investigation.

**Figure 2:**
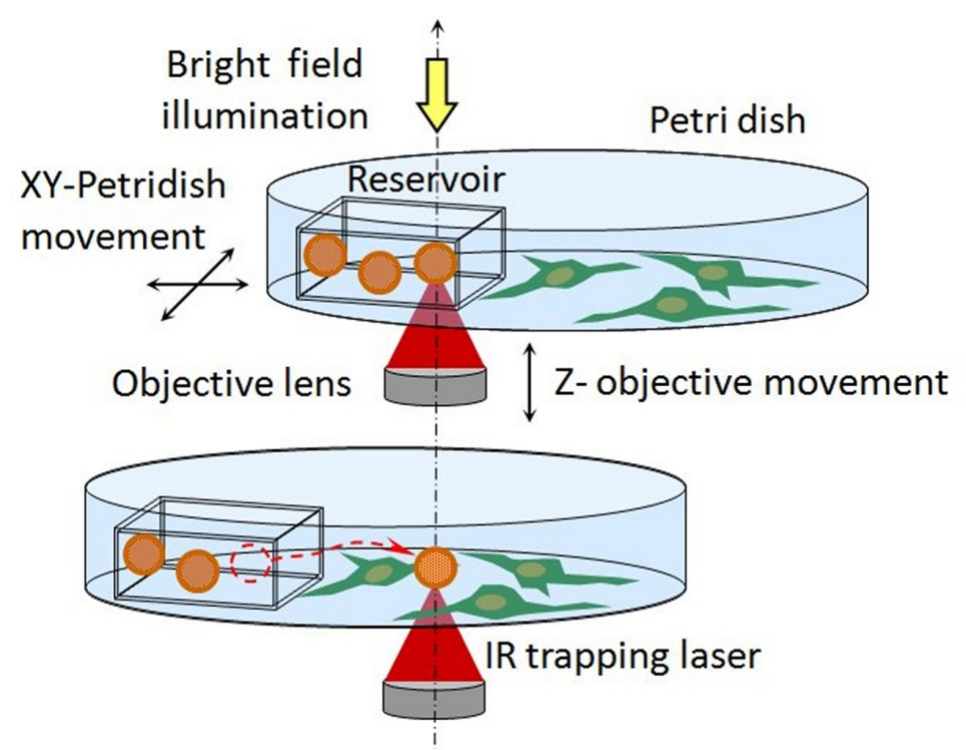
Scheme of local delivery by optically manipulated vectors carrying active molecules (adapted from [17]). A capillary reservoir, prefilled with vectors (e.g., coated microbeads, quantum-dot, or liposomes), is laid on the bottom of the Petri dish and a single vector is optically trapped by the IR laser. Moving the Petri dish stage in X-Y directions and the objective stage in Z direction, the vector can be extracted from the reservoir and positioned near or on the cell of interest with sub-micrometric precision.

Optical manipulation can considerably change the situation. Small reservoirs (e.g., thin square capillaries with inner dimensions of 25 μm × 25 μm) can be filled with micro or nano-vectors carrying active molecules in a physiological solution and laid on the bottom of the Petri dish (Figure 2). Due to capillarity, the vectors naturally remain inside the reservoir. However, single vectors can be trapped by IR-OT, transferred out of the reservoir, and positioned in contact or in proximity to the cell under observation.

**Figure 3:**
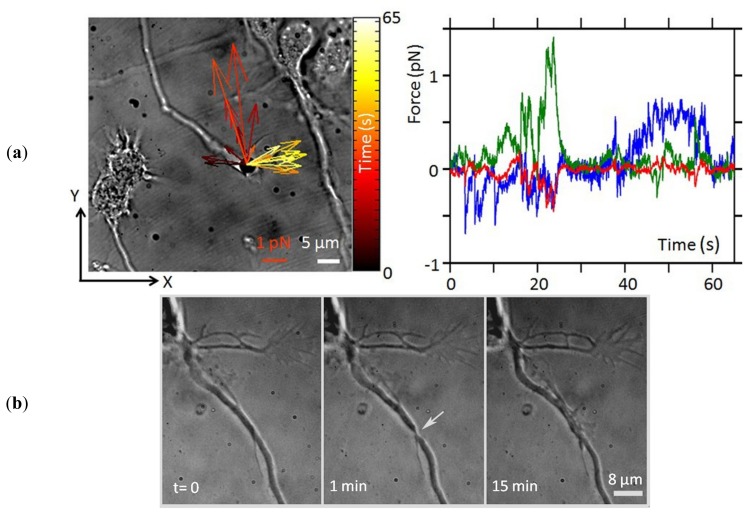

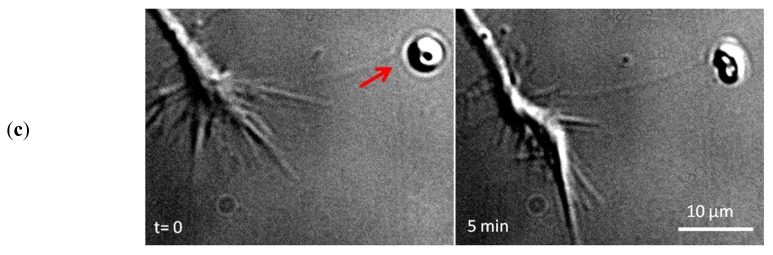
Examples of single-neuron experiments performed using optical manipulation techniques (experiments performed in the authors laboratories, unpublished data). (**a**) GC motility against an obstacle. Left panel: a vectorial representation of the force exerted by the cell on the obstacle mimicking an extracellular matrix component with calibrated stiffness (4-μm diameter silica bead trapped with an optical stiffness of 12 pN/μm in the x and y direction, and of 7 pN/μm in the z direction). Vector colors indicate the temporal sequence of forces produced by the cell on the optically trapped probe. Right panel: the corresponding force traces recorded by interferometric tracking of the trapped bead (sampled at 2 kHz). Blue, green and red traces are the forces measured in the x, y, and z coordinates, respectively; (**b**) The axon of a mouse hippocampal neuron (2 days *in vitro*) ablated with an UV laser (average power 2.5 μW, pulse rate 100 Hz, pulse length 400 ps). The white arrow indicates the ablation site. The healing of the axon, with the formation of a lamellipodium at the site of lesion is shown in the last slide; (**c**) Local delivery of SEMA3A molecules to the GC of a hippocampal neuron in a dissociated culture. SEMA3A molecules were encapsulated in lipid vesicles. A single vesicle (arrow) was positioned close to the GC by the OT and the content delivered by breaking the vesicle with a train of UV laser pulses. Two frames are reported: before (*t* = 0, **Left panel**) and 5 min after vesicle disruption and consequent release of molecules (**Right panel**). Retraction of the GC in response to the released SEMA3A molecules can be observed.

Some examples of single neuron experiments performed using optical manipulation techniques are shown in Figure 3. An optically trapped bead can be used as a probe to measure the small forces exerted by different compartments of the cell under controlled stimuli or to apply small forces to the cell. An example of a force measurement that was made to study growth cone (GC) motility is shown in Figure 3a. The technique allows the measurement of forces smaller than 1 pN, with 10–20 fN sensitivity, exerted by the GC in x-y-z. Moreover, the field of forces can be reconstructed over 1 min with sub-ms time resolution, allowing a detailed investigation. Ablating the axon allows observation of the regeneration process (Figure 3b), which involves the formation of a lamellipodium at the site of the lesion by 15 min post-injury. Local delivery of the repellent Semaphorin 3A (SEMA3A) protein molecules is illustrated in Figure 3c. The molecules encapsulated in a liposome, are released near the GC and induce GC turning.

In the next sections of this review, we will focus on the following optical manipulation approaches for cell signaling experiments: apply and sense pN forces, locally deliver active molecules, and laser nano-ablate the cells.

## 2. Apply and Sense Local Forces in Single-Cell Experiments

The optical potential of an optical trap can be well approximated by a harmonic potential. Consequently, the trapping force, *F,* experienced by the trapped particle varies linearly from the center of the trap: *F = k* × *x*, where *k* is the trap stiffness (a constant that can be determined by tracking the motion of the bead in the trap [9]) and *x* the distance from the center of the trap. Typically, the stiffness, *k*, of an optical trap is in the range of 10^−4^–1 pN nm^−1^ [18]. For a given particle, the trap stiffness can be controlled by changing the power of the trapping laser. Trapping a silica microbead generally requires a laser power of approximately 3 mW [19]. An optically trapped bead can be used as a force probe in a similar way to a cantilever in atomic force microscopy (AFM) or a magnetic probe in magnetic tweezers (MT); this is called photonic force microscopy (PFM) [20]. The first scanning probe image demonstrated for the outer surface of a small neurite of a cultured rat hippocampal neuron showed 40 nm axial resolution and 200 nm lateral resolution (limited by the probe size) [21].

The thermal position fluctuations of the probe in PFM are larger than those of an AFM cantilever. This behavior is advantageous when studying properties of the plasma membranes of living cells at the nanometer scale. For instance, measuring the local diffusing coefficient of the cell membrane has provided new ways to characterize structures with known properties, such as lipid rafts [22]. Furthermore, the technique can be used to determine the elasticity of the lipid bilayer and the binding properties of membrane components to the cytoskeleton [23].

Direct measurement of the forces generated by actin filament polymerization using an optical trap revealed that load forces of approximately 1 pN are sufficient to stall the growth of an eight-filament bundle, consistent with the theoretical model [24]. These results suggest that force generation by small actin bundles is limited by the dynamic instability of single actin filaments; therefore, living cells must use actin-associated factors to suppress this instability to generate substantial forces through the elongation of parallel bundles of actin filaments. Filopodia are thin cell extensions that sense the environment. They play an essential role during cell migration and cell–cell or cell–matrix adhesion by initiating contacts and conveying signals to the cell cortex. Because they are generally formed by bundles of a relatively small number of actin filaments, filopodia provide a model of choice to study elementary events in adhesion and downstream signaling. The forces exerted by the filopodia and lamellipodia of the GC of dorsal root ganglia (DRG) neurons have been measured with millisecond temporal resolution by using a trapped bead placed in front of the GC as a force probe [25]. A single filopodium exerts forces below 3 pN, whereas lamellipodia can exert forces up to 20 pN. Introducing specific inhibitors showed that no force was produced in the absence of actin polymerization. The elementary events underlying force generation in the lamellipodia of DRG neuronal cells have been described by studying the Brownian fluctuations (amplitude and frequency jump) of an optically trapped bead sealed on the lamellipodial membrane while using actin and myosin II inhibitors applied in bulk [26]. Long-term tracking, with sub-millisecond resolution, of a bead attached to a neuron while preserving sub-nanometer sensitivity in a spatial range of centimeters has been demonstrated [27]. The authors showed that the application of small constant forces (<1 pN) for a long time (~30 min) influenced GC motility. The suitability of the system has been further tested by time-modulating the force-clamp condition to study the role of statically and dynamically applied forces in neuronal differentiation. The forces that stall the retraction of a single filopodium have also been measured by using beads with different coatings [28]. The results suggest that the number of receptor–ligand interactions at the filopodial tip determines the maximal retraction force exerted by filopodia and that a discrete number of clustered receptors is sufficient to induce high retraction stall forces.

Axonal regeneration has recently been studied using force spectroscopy of an optically trapped bead attached to the axonal membrane before and after axonal ablation [29]. Axonal adhesion to the substrate and the viscoelastic properties of the membrane during regeneration were measured, and the subsequent axonal regeneration was documented by long-term live imaging. This study demonstrated that brain derived neurotrophic factor (BDNF) regulates neuronal adhesion and favors the formation of actin waves during regeneration after axonal lesioning. The possibility of using holographic OT to probe the forces at more points of the sample cell has been proposed [30]. This technique has been employed to measure mechanical coupling between *Aplysia* bag cell growth cones and beads functionalized with the neuronal cell adhesion molecule, apCAM. Another advanced optical manipulation technique was recently demonstrated in a study of nerve fiber growth [31]. An optically driven birefringent bead and circularly polarized light were used to create a localized microfluidic flow that generated a small sheer force, of approximately 0.2 pN, to direct the GC.

Interestingly, optical stiffness and force range of PFM are complementary to AFM and MT [32], thus allowing to design a large variety of single-molecule force spectroscopy techniques. Discussing single-molecule techniques is beyond the scope of this review, and some very good reviews are already available (e.g., [33]). Though many properties and functions of different bio-molecules can be revealed using these single-molecule techniques, an important criticism refers to the experimental conditions, which remain far from the physiological conditions of living cells. However, considerable progress has been made in this realm, and solutions have been proposed to overcome some of the challenges, as recently appraised by Oddershede [34]. Manipulating individual molecules requires a force-transducing handle that binds specifically to the system of interest. The handles typically utilized for optical manipulation are silica/polystyrene and metallic beads. Promising force-transducing handles for single-molecule optical manipulation in living cells are quantum dots (QD) [35] or gold nanorods [36]. A single QD or gold nanorod can be optically manipulated, and their positions can be tracked with high resolution at certain locations due to their fluorescence quantum yield.

Micro- and nano-sized beads, which are largely used in optical trapping, can be easily functionalized with almost any type of protein, allowing for the combination of mechanical and chemical local stimulation of the cell. This allows for the extracellular attachment sites to be mimicked with different, yet controlled, stiffness and study signal transduction. For instance, using fibronectin-coated beads positioned on fibroblasts and trapped with different strengths, proportional strengthening of the cytoskeletal linkages was demonstrated [37]. Local force or geometry sensing are transduced into biochemical signals that result in cellular responses, even for complex mechanical parameters, such as substrate rigidity and cell-level structures. These responses regulate cell growth, differentiation, shape changes and cell death [38]. Cells rapidly transduce forces exerted at extracellular matrix (ECM) contacts into tyrosine kinase activation and cytoskeletal proteins recruitment to reinforce the integrin–cytoskeleton connections and to initiate the formation of adhesion sites. The relationship between these two processes was studied at the sub-micrometric scale using optically manipulated vectors (coated beads) [39], allowing the demonstration that talin 1 is critical for the force-dependent reinforcement of the initial integrin-cytoskeleton bonds, but not for tyrosine kinase activation. Many aspects of cellular motility and mechanics are cyclic in nature, such as the extension and retraction of lamellipodia or filopodia. These mechanical cycles induce the production of mechano-chemical signals that can alter long-term cell behavior and transcription patterns. Stretching can alter physical properties or sites exposed to matrix molecules as well as internal networks; thus, cell contractions can cause a secondary wave of mechano-regulated outside-in and internal cell signal changes. A basic concept of the mechano-chemical cycles and the ways in which they can be described and understood was developed and discussed by Vogel and Sheetz [40].

Biomechanical approaches were recently applied to understand tumor invasion [41]. When a cell is mechanically stressed, forces are transmitted through the cytoskeleton and might stress the nucleus triggering gene transcription. As a result, cyto-adherence is altered and different patterns of gene expression are invoked, leading to changes in cell motility. Local mechanical effects are thus thought to play a significant role in stimulating tumor cells to become invasive [42]. It has been demonstrated that even though tumors are relatively stiff due to their ECM, tumor cells themselves are elastically softer [43]; consequently, they are able to move through a dense ECM. There is a continuous biomechanical interaction between cells and the ECM, leading to adaptations of cell motility [44]. Therefore, the hypothesis that cellular biomechanics may play a significant role in tumorigenesis and cancer cell invasiveness is gaining increasing support [45]. Different optical techniques have been used to determine the mechanical properties of living cells, such as generating rigidity gradients using multiple OT [37] and deforming cells flowing in a fluidic channel with an optical stretcher [46].

Small surface areas or entire cells can be mechanically probed with forces from 0.1 to 100 pN and displacements ranging from 1 nm to 10 μm. The force strength and the specific technique needed to obtain a response depends on the cellular mechanism under study [47]. To induce significant conformational changes of force-transmitting proteins, the force required to break the bond between two proteins is the realistic upper limit of the force required. The fibronectin–integrin bond strength, for instance, was experimentally determined to be in the range of 30–100 pN [48]. In contrast, the minimum external force required to induce an intracellular change at the single molecule level can be estimated by considering thermal fluctuations. To change intracellular biochemical reaction rates, the effect of the external force on the protein conformation must overwhelm that associated with thermal fluctuations. Considering that the thermal energy, *k_B_T* = 4 pN nm (where *k_B_* is Boltzmann constant and *T* is the local temperature expressed in Kelvin) and that protein conformational changes are in the range of 1–10 nm, the required force levels are in the range of 0.4 to 4 pN. Another important consideration when studying cellular mechanics are the spatial and temporal resolutions. Most mechanical studies of the cytoplasm treat it as a uniform viscoelastic fluid; therefore, the reported viscosity values vary by four-orders of magnitude [49]. A regional approach to cellular mechanics that considers the differences between the properties of different cellular compartments is therefore highly important [50]. Optical manipulation techniques permit local mechanical measurements of the cytoplasm and cell surface over short time periods; therefore, they are biologically well suited for studies of cytoplasmic mechanics. MT and OT have a higher sensitivity than AFM for detecting small forces, such as tens of pN, and are therefore more useful for viscoelastic studies of the localized regions of the cell membrane. Moreover, these techniques allow for the simultaneous detection of deformations induced by multiple probes at different sites of the cell. Trapping and manipulating a microbead to get in contact with the cell membrane allows pulling a thin tether membrane. Video or interference tracking of the bead positions provides the force-elongation curve from which the local viscoelasticity can be determined and models for tether formation can be formulated [51]. Different membrane tether models have been reported for calculating the bending modulus and the surface membrane tension from the force-elongation curves of various cell types, including neurons [52], outer hair cells [53], breast cancer cells [54], fibroblasts [55], and human mesenchymal stem cells [56]. The force resulting from actin polymerization and depolymerization was recently measured in a cell by monitoring the restoring force of the plasma membrane at the end of a membrane tube [57]. The detection of a pulling force associated with depolymerization implies that the membrane is attached to the actin bundle and the filaments are not always treadmilling. Further studies are necessary to establish if this pulling force contributes to cell contraction during cell motility.

## 3. Local Delivery

Cell signaling is characterized by strict spatial and temporal control of chemical interactions [58], which is achieved by specific compartmentalization of different machineries. The specific subcellular expression of receptors and enzymes and fine control of their chemical affinities with ligands and substrates [59] achieves specificity in pathway activation. Therefore, a detailed study of cell signaling processes requires subcellular manipulation [60] and single-molecule approaches [33]. Properly mimicking the process of chemical release that characterizes the fast and short-distance diffusion of small volumes in confined areas is particularly important for studying neuronal cells, which have a complex morphology and possess specific structures for information processing and signal transmission (axons, dendrites, spines, and synapses).

Different tools have been developed with the aim of delivering chemical stimuli at high spatial and temporal resolution, including puffers and microejectors [61] for constant pressure delivery of nano to pico-liter volumes. However, these tools feature limited control over the released volume, low spatial precision and the risk of leakage from the micropipette. The photoactivation of caged compounds [62] has many advantages over the former delivery techniques: it allows both extracellular and intracellular stimulation; the gating time of delivery is rapid, ranging from sub-microseconds to milliseconds; and the release location is restricted to the area of incidence of the uncaging light [63]. UV-uncaging is a very popular tool for focal stimulation of single synaptic sites, but it suffers some major drawbacks. Because the synthesis of caged molecules is complex, large active molecules, such as peptides or proteins, cannot be caged. Moreover, most caged compounds were observed to produce undesired side effects, such as blocking both glycine and GABA_A_ receptors [64]. A delivery precision comparable to that of two photon uncaging is obtained by releasing the contents of microcontainers [65]. Microcontainers, or physical cages, are nano- to micro-sized capsules synthesized from different materials [66]. The advantages of using physical cages are the ability to encapsulate a variety of molecules and the possibility of moving and dissecting single loaded capsules using optical techniques [11].

Nanocapsules have been synthesized using various materials, such as polystyrene−acrylic or silica, by the formation of colloidal silver nanoparticles coated with silica and subsequently silver-etched [66]. These capsules were loaded with carbachol that was released by laser pulses onto single CHO cells expressing muscarinic receptors. Polylactic-co-glycolic acid (PLGA) capsules were also synthesized for loading chemotactic factors and were used to establish persistent concentration gradients at the micrometric scale [67]. Among the physical cages, lipid vesicles are very convenient tools for chemical encapsulation. Due to their prominent use in drug delivery [68], a variety of techniques have been developed to fine-tune their physical and chemical characteristics, such as their dimension, surface charge and chemical functionalization. Lipid vesicles have been employed for single-cell stimulation [69]. Single unit electrical stimulation has been obtained in hippocampal neuronal cultures by optical manipulation and photolysis of micro-sized KCl-containing vesicles [17]. Due to the small volume of solution enclosed in the lumen of the vesicles (femtoliters for micro-sized vesicles), encapsulation in lipid vesicle is a way to control the amount of a compound delivered. Therefore, details of the transduction pathways involved in processes such as neuronal guidance can be more easily obtained. By carefully evaluating the amount of a substance that is released, one can quantitatively correlate morphological responses and the intermediate steps of a signal transduction pathway [70].

Encapsulation of single molecules in lipid vesicles has also been exploited for monitoring single-protein folding [71] or for performing spectroscopy of a single biomolecule [72]. Quantitative assessment of encapsulation efficiency (ratio of the concentration of the encapsulated molecule to the bulk concentration) is crucial for single molecule approaches. Different methodologies have been proposed to address this issue. Confocal detection with single-molecule sensitivity associated with numerical calculations based on a three-dimensional diffusion equation allows the number of molecules released from vesicles immobilized in an optical trap to be calculated [73]. Fluorescence correlation spectroscopy has also been used to measure vesicle contents and to demonstrate that the number of molecules encapsulated in lipid vesicles is a random variable following a Poisson distribution [74]. Based on this assumption, the encapsulation efficiency can be evaluated using QD by measuring the fraction of empty vesicles for different concentrations of encapsulated QD [70].

Another technique for local chemical stimulation of specific biochemical pathways is optical manipulation of beads functionalized with signaling molecules. This technique has the advantage of avoiding any spillover of ligand from the site of contact with the bead because the molecules of interest are covalently attached to the bead surface. In particular, neurotrophins have important roles in neuronal development, and cellular responses strongly depend on the localization of chemical stimulation because neurotrophin receptors are differentially expressed on the neuronal cell surface.

Polystyrene beads coated with nerve growth factor (NGF) were first used for local stimulation of DRG growth cones [75]. Recently, beads coated with a single BDNF molecule were optically delivered to different compartments of neuronal cells, and the activation of signaling pathways was confirmed by the occurrence of TrkB phosphorylation, long-lasting activation of calcium signaling at the soma, and c-Fos nuclear signaling [76]. Gradients of BDNF, generated by slow-release from beads placed near geniculate axons, were utilized to assess the critical period during which BDNF is the attractant [77]. Microbeads functionalized with ligands were also used to test single molecule interactions with receptors [78]. Ligands were bound to specific receptors, and picoNewton forces were applied through the OT to induce bond rupture. This method was recently exploited to study the role of endocytosis in enhancing the binding strength of Dll1-Notch during Notch signaling. Beads functionalized with recombinant Notch1 protein were presented to Dll1-expressing cells, and the bond strength was measured using OT [79]. Similarly, the forces involved in virus–host cell binding were investigated using OT and AFM-based single-molecule force spectroscopy using polystyrene beads coated with influenza A X-31 virions, by measuring the interactive forces between the viruses and living cells [80].

OT have also been used in combination with microfluidic devices for local control of the cellular environment and single-cell manipulation. Indeed, microfabrication techniques allow production of micron or sub-micron scale structures with complex geometries, which can be used for precise control of chemical fluxes and the delivery of soluble factors [81–83]. On-chip single-cell assays were first developed to analyze bacterial adaptation processes when the nutrient concentration changed [84] and have been used for cell-sorting [85]. A microfluidic device integrated with OT was designed to allow precise and reversible changes in the glucose concentration around single *Saccharomyces cerevisiae* cells in less than two seconds [86]. Cytometry using multiple refractive OT combined with microfluidics and optical microscopy was developed to expose yeast cells to reagents in a controlled manner and analyze their responses using fluorescence microscopy [87]. Recently, a fully integrated system relying on miniaturized fiber-based OT has been developed for microfluidic flow cytometry based on Raman-spectroscopy or fluorescence analysis [88]. A multifunctional system has also been developed for simultaneous spectral analysis of biochemical contents and electrophysiological reactions in single cells [89], and the recruitment of vinculin in single endothelial cells upon the application of external forces via OT has been investigated using a microfluidic platform [90].

## 4. Laser Dissection

Living tissues present a complex architecture of multiple cell types embedded in ECM with chemical, mechanical and topographical features localized on nanometer to micrometric scales [91]. Cell–cell and cell–matrix contacts influence cytoskeletal architecture, cellular metabolism and cell survival, producing clusters of distinct cell types with specific roles in the physiology of the organ [92]. Furthermore, the ECM is a three-dimensional network surrounding all the cells in the body, which functions as a biophysical filter for the protection, nutrition and innervation of cells and as a biophysical medium that facilitates immune responses, angiogenesis, fibrosis and tissue regeneration [93]. The ECM is remodeled by cells during various physiological processes, such as embryonic development, tissue growth and the synaptic plasticity of the adult brain [94], as well as during pathophysiological events such as the progression of glioma.

In this context, another optical manipulation technique, laser dissection, has gained popularity because of its ability to access intricate and dense environments and locally alter extracellular, cellular, and sub-cellular sites. Laser dissectors based on pulsed laser sources produce high peak energies of densely packed photons at a low average power. This peculiarity allows laser dissectors to overcome the breakdown energy of a material, eliciting ablation in a confined, extremely small volume. Berns *et al.* [14] exemplarily explained in their seminal work that: “*Although in optical microscopy the imaging resolution is diffraction-limited, diffraction is not necessarily a limiting resolution factor for laser ablation. It occurs whenever the energy delivered to the sample is higher than the break-down energy threshold of the irradiated material.*” Indeed, tight focusing of Gaussian laser beams can generate high photon flux, and the accurate control of the delivered laser power can modulate the dimension of the “hot spot” within the diffraction-limited focal volume. Distinct pulsed laser sources have been employed to perform laser dissection at the UV-VIS to the IR wavelength range, with pulse duration ranging from a few nano-seconds to femto-seconds. For further details on the requirements of laser microsurgery instrumentation, how the field grew with the evolution of pulsed laser systems, and a comparison of experimental achievements of several laboratories using specific laser sources, refer to the review of Magidson *et al.* [95]. In the subsequent section, we will review some applications of laser dissection in single-cell and *in vivo* studies that preserve the control, precision and repeatability of the light nano-scalpel used for molecular biological studies.

The cell is the functional unit of living tissue, and its plasma membrane delimits the internal organization and constitutes a barrier that selectively controls communication with the extracellular environment. Delivering low-energy optical pulses to the cell membrane in a small window of time (on the order of a few milliseconds) generates reversible spatially confined ablation, which is called photoporation. Photoporation enables intracellular delivery of substances [96], such as QD gold nanoparticles, DNA, RNA, or fluorescent markers, that would otherwise require carriers (e.g., viruses or liposomes), or invasive approaches (e.g., patch-electrodes or electroporation) to bypass the membrane. This method has been applied mainly to deliver exogenous DNA or RNA [97], thereby allowing targeting of specific single cells with high transfection efficiency. Moreover, because of its focal transfection capability, photoporation allowed the role of a particular mRNA in different regions of living neurons [98] (*i.e.*, in a dendrite or in the cell body of primary rat neurons) to be evaluated. Recently, photoporation was used to introduce biomolecules into the cells of living animals, thus providing an alternative to genetic manipulation of developing embryos via functional modulation of individual cells under sterile conditions [99]. To achieve single-cell photoporation in thick non-transparent tissues, the use of optical fibers in conjunction with a miniaturized microfluidic system for localized drug delivery has been proposed, paving the way for the clinical application of this nanosurgical tool [15]. However, the microfluidic system requires a considerable number of molecules and does not allow control over the number of delivered factors.

Moving our attention from the cell surface to the intracellular architecture, laser dissection can be used to selectively destroy organelles and supramolecular structures, thereby applying a knock-down approach for elucidating the role of the ablated compartment. Laser dissection of chromosomal regions in living cells was performed to study the role of secondary constrictions as nucleolus organizers [100]. Laser ablation of the centrosomes did not preclude the formation of a mitotic spindle in mitotic cells [101] or axonal extension in developing hippocampal neurons [102].

At the whole cell level, laser-induced cell fusion was used to generate viable hybrid cell-types [103]. Extraction of tissue regions allowed local proteomic/genomic screening to be applied or the creation of type-defined cell cultures [104]. The separation of subcellular compartments as the distal and proximal parts of a dissected axon elucidated the role of anterograde or retrograde vesicular transport in the formation of a new growth cone [105].

Several laboratories use laser dissection systems as a complementary approach to pharmacological and genetic inhibition of single proteins because they allow selective manipulation of whole organelles and structures with high spatial resolution [106]. However, one of the more challenging applications of laser dissection is the study of wound healing and the regeneration processes of injured tissues. Indeed, the precision of the light scalpel allows the development of reproducible experimental models of injury with which to test genetic and pharmacological treatments that favor regeneration of the damaged tissue. For example, laser micro-irradiation can be used to induce focal damage in the nucleus for analysis of the recognition of damaged DNA [107] and the repair process that counteracts the consequences of DNA lesions [108]. Defects in signaling DNA damage or repairing damaged DNA contribute to aging and various disorders, including developmental defects, neurodegenerative diseases, and cancer [109]. Another field of study is the understanding of molecular mechanisms involved in neuronal regeneration. Neurons of the peripheral nervous system (PNS) have an intrinsic ability to regenerate after axonal injury, whereas neurons in the central nervous system (CNS) lack this regenerative capacity [110]. Thus, understanding the molecular mechanisms involved in the intrinsic ability of an axon to re-grow is crucial for developing new strategies that promote nerve regeneration. Regeneration of damaged neurites can be impaired by collateral damage in the surrounding cells, and pharmacological approaches applied to whole tissues can be misleading because of the distinct reactions of the neighboring cell-types [111]. Therefore, several *in vitro* systems based on laser dissection have been developed to study the regeneration process under controlled environmental conditions [112]. A laser microdissector can induce localized, controlled [113] and reproducible damage to the neurite without affecting the integrity of any coating of the culture support, which could hamper axonal re-growth. Recently, an *in vitro* model using mouse hippocampal neurons demonstrated that CNS neurons are able to regenerate their axons (after a laser-induced lesion) during the first 3 days *in vitro*, but not later [29]. The mouse neuronal experimental model provides the opportunity to identify the role of various molecules that may affect neuronal regeneration trough the use of knock-out and transgenic mice.

To identify the role of single neuronal connections, laser ablation in combination with fast calcium imaging has been used to obtain an *in vitro* model of neuronal network injury for investigation of the changes in the electrophysiological activity and functional connectivity of the network with subcellular resolution [114]. Generally, *in vitro* models poorly resemble the *in vivo* scenario and thus it is necessary to confirm the obtained results *in vivo*. Axonal regeneration studies using laser dissection systems have been conducted with organism models such as *Caenorhabditis elegans* and *Drosophila* [115] or with the cortical neurons of living mice [116]. Moreover, laser dissection have been exploited to induce vascular disruptions within the parenchyma of the rat brain to obtain a model of local cortical ischemia [117].

The critical step of the axonal regeneration process is the assembly of a new navigating GC capable of guiding the axon to its proper target [118]. Furthermore, it has been shown that the formation of GC like structures [119] that travel along the injured neurite has a role in the regeneration process [29]. GC is the sub-cellular compartment at the tip of an extruding neurite that contains all of the molecular machinery necessary for cellular motility and sensing the mechanical and chemical properties of the ECM. Understanding how ECM properties influence the motility of the GC could provide essential information for the design and construction of implantable scaffolds that would support tissue regeneration. In this context, laser dissection is an invaluable tool for producing the prototypes of micropatterned planar adhesion substrates with the appropriate geometrical [120] and chemical features [121] or biocompatible three-dimensional scaffolds with microchannels that direct cell growth [122].

Laser dissection in combination with OT constitutes a nano-workstation that offers the possibility of quantifying the cytoskeletal dynamics of wound healing [123], dissecting GC dynamics at a molecular level [25], or achieving completely contact-free manipulation of developing embryos [124].

## 5. Concluding Remarks

**Figure 4:**
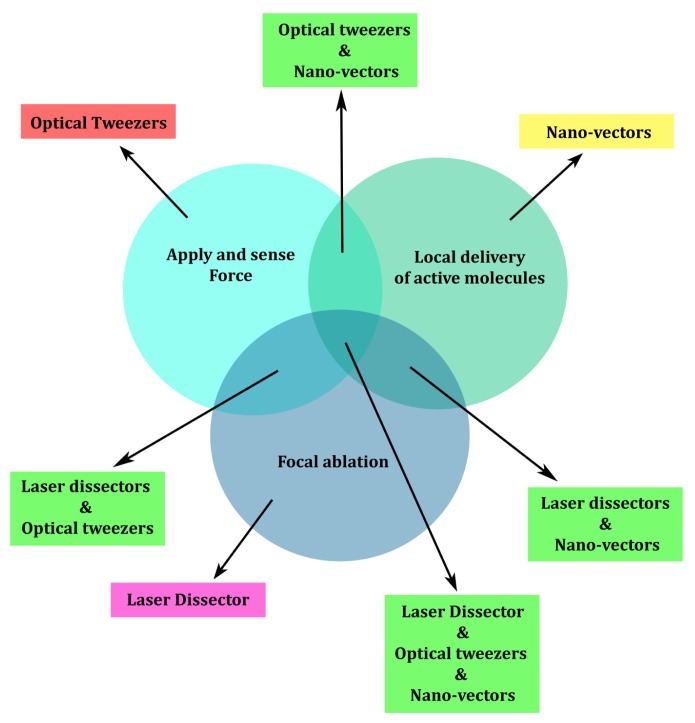
Optical manipulation techniques and their interdependencies for optically driven cell signaling experiments. The intersecting blue circles indicate the desired experimental goals, and the arrows indicate the required techniques to accomplish the goals.

Cell signaling driven by optical manipulation has been discussed in previous sections, focusing on three main techniques: application and probing of pN forces, local delivery of small amounts of active molecules, and laser nano-ablation. As illustrated schematically in Figure 4, these three techniques can be applied separately or jointly, increasing the range of their applications. Starting from the experimental capabilities of each approach, the simultaneous application of the three techniques allows a precise and reliable stimulation protocol at the level of a single cell, addressing very small areas of its sub-cellular compartments. For instance, the use of micro- and nano-vectors in conjunction with optical manipulation and laser photolysis allows scaling down the stimulation to a few molecules, localizing their action to areas of hundreds of square nanometers on the cell membrane and following their internalization and the cell’s response. Laser ablation allows to photoporate the cell membrane and thus bypass it to investigate only the intracellular effects of a molecule, or to locally release physically caged compounds. OT enable arbitrary selection of the cell to monitor, the location and time of local stimulation, offering the unprecedented possibility of combining mechanical and chemical cell stimulation.

Considering the complex nature of cell signaling mechanisms, optical manipulation techniques can considerably contribute to reducing the uncertainty in experimental protocols and data interpretation by avoiding activation of collateral cell pathways, thus allowing the investigation of cell signaling at a single molecule resolution.
